# Binding to medium and long chain fatty acyls is a common property of HEAT and ARM repeat modules

**DOI:** 10.1038/s41598-019-50817-6

**Published:** 2019-10-02

**Authors:** Tie-Mei Li, John P. Coan, Krzysztof Krajewski, Lichao Zhang, Joshua E. Elias, Brian D. Strahl, Or Gozani, Katrin F. Chua

**Affiliations:** 1 Department of Medicine, Stanford University School of Medicine, Stanford, CA 94305 USA; 2 Department of Biology, Stanford University, Stanford, CA 94305 USA; 3 Program in Cancer Biology, Stanford University School of Medicine, 291 Campus Drive, Stanford, CA 94305 USA; 4 Department of Biochemistry & Biophysics, University of North Carolina School of Medicine, Chapel Hill, NC 27599 USA; 5 Chan Zuckenberg Biohub, Stanford, CA 94305 USA; 6 Geriatric Research, Education, and Clinical Center, Veterans Affairs Palo Alto Health Care System, Palo Alto, CA 94304 USA

**Keywords:** Protein-protein interaction networks, Acetylation

## Abstract

Covalent post-translational modification (PTM) of proteins with acyl groups of various carbon chain-lengths regulates diverse biological processes ranging from chromatin dynamics to subcellular localization. While the YEATS domain has been found to be a prominent reader of acetylation and other short acyl modifications, whether additional acyl-lysine reader domains exist, particularly for longer carbon chains, is unclear. Here, we employed a quantitative proteomic approach using various modified peptide baits to identify reader proteins of various acyl modifications. We discovered that proteins harboring HEAT and ARM repeats bind to lysine myristoylated peptides. Recombinant HEAT and ARM repeats bind to myristoylated peptides independent of the peptide sequence or the position of the myristoyl group. Indeed, HEAT and ARM repeats bind directly to medium- and long-chain free fatty acids (MCFA and LCFA). Lipidomic experiments suggest that MCFAs and LCFAs interact with HEAT and ARM repeat proteins in mammalian cells. Finally, treatment of cells with exogenous MCFAs and inhibitors of MCFA-CoA synthases increase the transactivation activity of the ARM repeat protein β-catenin. Taken together, our results suggest an unappreciated role for fatty acids in the regulation of proteins harboring HEAT or ARM repeats.

## Introduction

Acyl groups ranging from acetyl to long-chain fatty acyls covalently modify proteins for various biological purposes^1,2^. For example, lysine acetylation of histone proteins regulates chromatin in a highly dynamic manner, in part via the sensing and transduction of the acetylation event by chromatin-regulatory proteins that harbor reader domains^3,4^. Lysine acetylation can be added or removed by lysine acetyltransferases and lysine deacetylases respectively, while acetyllysine-binding domains (or readers) couple lysine acetylation events to various downstream biological effects. In addition to lysine acetylation, many structurally diverse acyl modifications have been identified on histones and other proteins^2,5^. Some of the enzymes that catalyze lysine acetylation also catalyze other short chain lysine acylations such as crotonylation, succinylation and propionylation^5^. Moreover, specific lysine deacetylases, at least *in vitro*, show more robust activity on long chain acyl substrates^6,7^. Reader domains for short chain acylation such as crotonylation and succinylation have been identified^8–11^. These studies suggest that analogous to the paradigm of lysine acetylation signaling, reader domain proteins exist for the broader family of acyl modifications. However, the identity of acyl modification binding proteins is largely unexplored.

Here, we report the identification of HEAT and ARM repeats as reader domains of medium- and long-chain acylation. Characterization of the interaction suggests that fatty acyl-binding is a common property of diverse HEAT and ARM repeat proteins. We also provide functional evidence that β-catenin function, via its ARM-repeats, is impacted by the interaction with fatty acids in cells.

## Results

### Identification of HEAT and ARM repeats as potential protein myristoylation readers

**Figure 1:**
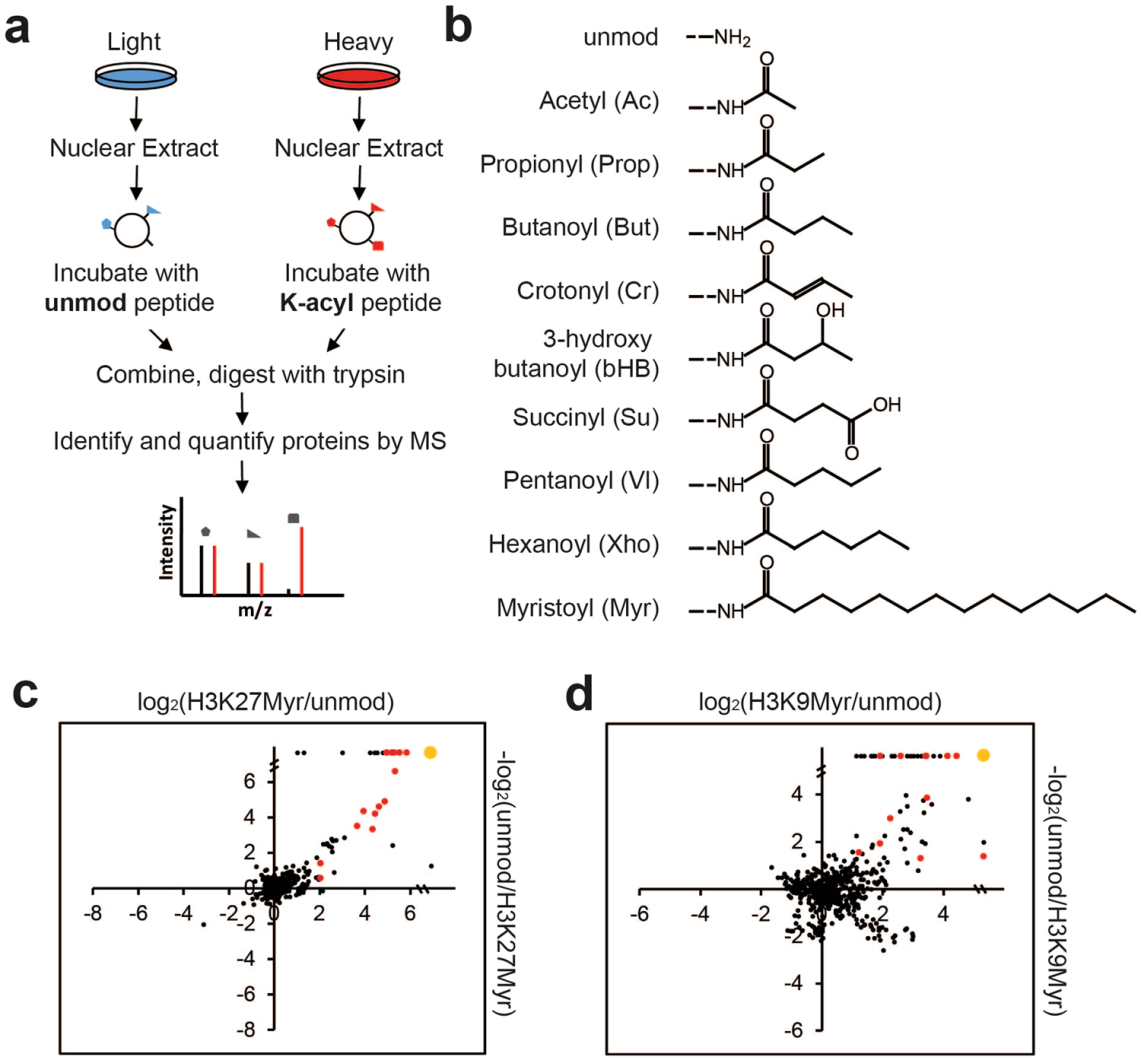
Identification of the binding candidates of protein acyl modifications. (**a**) SILAC (Stable Isotope Labeling by/with Amino acids in Cell culture) and peptide pull-down experiment design. Each pair of peptide pull-downs were repeated twice, through forward (unmodified-light, acyl-heavy) and reverse (unmodified-heavy, acyl-light) experiments. (**b**) Structures of unmodified (unmod) and acyl-modified lysine side chain of the peptides used in this study. (**c** and **d**) Scatter plots showing protein quantification results of forward (x-axis) and reverse (y-axis) experiments for two lysine myristoylated peptides compared with the corresponding unmodified peptide. Red dots highlight proteins with HEAT or ARM repeats. The large orange dot on the upper right corner of (**c**,**d**) represents proteins (42 in H3K27myr experiment; 37 in H3K9myr) with no forward and reverse ratios reported by MaxQuant software but manually found enriched based on peptide signals, among which 23 and 14 proteins are HEAT or ARM repeat proteins respectively.

We combined peptide pull-downs (PPD) and a quantitative proteomic approach, Stable Isotope Labeling by/with Amino acids in Cell culture (SILAC)^12^, to identify potential binding proteins of lysine acylation (Fig. 1a). Histone H3 peptides with unmodified lysine or various types of acyl-modified lysine (Fig. 1b) were used as baits to pull down proteins from Hela cell nuclear extract that was labeled with either light (12 C and 14 N) or heavy (13 C and 15 N) lysine and arginine. Two groups of peptides with modifications on lysine 9 or lysine 27 of histone H3 were used as baits. For each combination of acyl-vs-unmodified peptide pull-down, two replicates were generated with reciprocal stable isotope labeling, i.e. forward (unmodified-light, acyl-heavy) and reverse experiments (unmodified-heavy, acyl-light). We employed high resolution liquid chromatography coupled tandem mass spectrometry to identify the proteins from the peptide pull-down experiments and MaxQuant software^13^ for protein quantification (Supplementary Tables S1 and S2, Fig. S1). Proteins that showed an acyl/unmodified ratio over two-fold in both the forward and reverse experiments were considered significantly enriched for preferential binding to the acyl modification state relative to the unmodified state.

Short acyl modifications (defined as having equal to or less than six carbon atoms, which include acetyl-, propionyl-, crotonyl-, butanoyl (butyryl)-, 3-hydroxyl butanoyl-, succinyl-, pentanoyl- and hexanoyl-) all resulted in less than ten enriched proteins being pulled down relative to unmodified control peptides (Supplementary Fig. S1 and Tables S1, S2), and most of the enriched proteins were either proteins with known reader domains such as the YEATS domains^9–11,14–18^ or known to associate with a YEATS domain protein. Surprisingly, compared to other acylated peptides, dozens of proteins were enriched in the myristoylated peptide pulldowns, with some of these proteins showing myristoyl/unmodified enrichment ratios of greater than 10-fold (Fig. 1c,d, Supplementary Table S3). Moreover, several proteins are enriched for binding to the myristoylated peptide in one replicate but do not have quantification ratios in the other. Manually checking these protein signals, we found that many were enriched in both replicates but the software MaxQuant failed to report the protein ratios. This occurs when the ratio is either zero or infinitely great due to a zero value in the unmodified peptide pull-down. To solve this problem, we manually identified the enriched proteins based on the peptide signals of a given protein from the missing ratio list (see method section for details). The full lists of enriched proteins are shown in Supplementary Table S3.

We next checked the Uniprot and Interpro databases for the annotated domains of the candidate myristoyl-binding proteins. An uncommonly large proportion of these proteins contain either HEAT or ARM repeats, two closely-related protein repeats characterized by their generally hydrophobic alpha-helical structures. Specifically, 27 out of 96 enriched proteins in the pulldown with an H3 peptide myristoylated on lysine 9 (H3K9myr) and 37 out of 80 enriched proteins with the pulldown with an H3 peptide myristoylated on lysine 27 (H3K27myr) contain HEAT or ARM repeats (Supplementary Table S3). This represents a 4.8- and 8.2-fold enrichment of HEAT and ARM proteins in the pulldowns as compared to the human proteome, respectively, with associated Chi-Square test *p*-values of 1.17E-21 and 5.83E-56 (Supplementary Table S4).

### HEAT and ARM repeats interact with the myristoyl moiety directly

**Figure 2:**
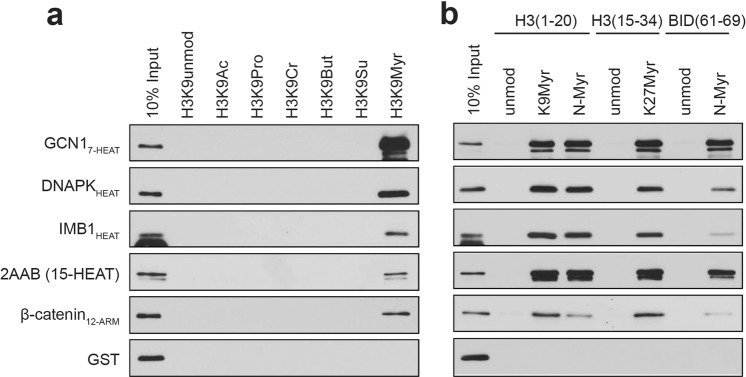
HEAT and ARM repeat proteins bind to myristoylated peptides directly. Peptide pull-down experiment using recombinant proteins purified from *E. coli* with (**a**) H3 peptides with different acyl modifications on lysine 9, and (**b**) peptides with a myristoyl group on a lysine side chain or the N-terminus. Full-length blots are presented in Supplementary Fig. S2.

We next tested whether HEAT and ARM repeat proteins directly bind to myristoylated peptides *in vitro*. We expressed HEAT repeats from five proteins, three that were positives in the proteomic experiments: GCN1 (Translational activator GCN1), DNAPK (DNA-dependent protein kinase catalytic subunit), and IMB1 (Importin subunit beta-1); one canonical, well-defined HEAT repeat: 2AAB (Serine/threonine-protein phosphatase 2 A 65 kDa regulatory subunit A beta isoform) and one canonical ARM repeat: β-catenin. *in vitro* binding assays showed that all of the recombinant GST (Glutathione S-transferase)-tagged proteins tested bound to H3K9myr peptides, and no binding was seen for shorter acyl-peptides (Fig. 2a, Supplementary Fig. S2). Myristoylation is most often found on the protein N-terminus^19^, so we also tested if the binding was dependent on the position of the myristoyl group within the peptide. An N-myristoylated H3 peptide largely retained the interaction with HEAT and ARM proteins (Fig. 2b, Supplementary Fig. S2). We used another pair of peptides derived from the protein BID, which is physiologically myristoylated on an internal glycine residue upon caspase cleavage^20^. Again, all proteins tested bound to myristoylated BID but not the unmodified BID peptide (Fig. 2b, Supplementary Fig. S2). Overall, all the HEAT and ARM repeat proteins that we tested bound to myristoylated peptides regardless of the underlying peptide sequence or position of the modification, suggesting that longer chain acyl-binding may be a general property of HEAT and ARM repeat proteins.

### HEAT and ARM repeat proteins exhibit distinct acyl-binding profiles

**Figure 3:**
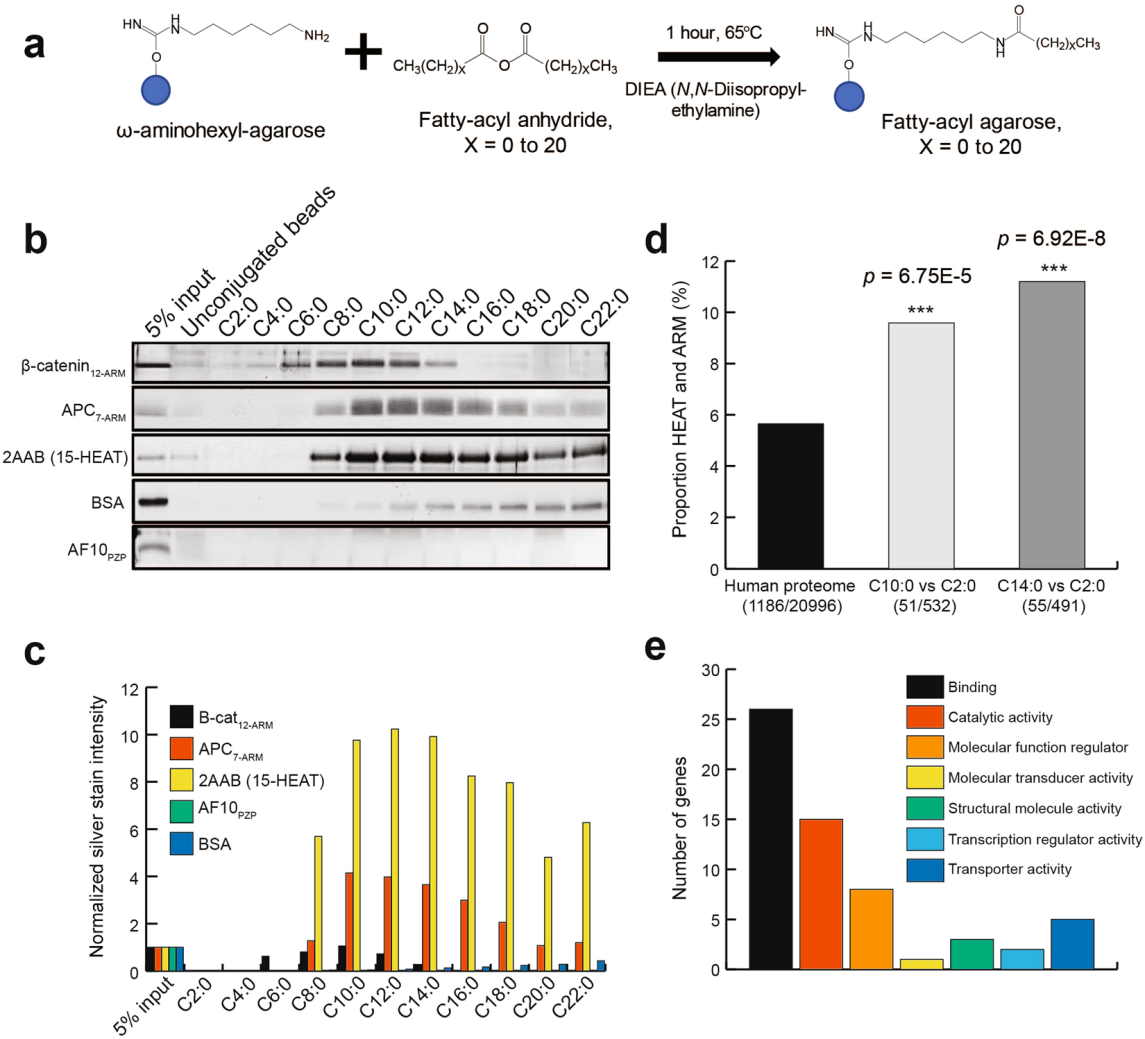
HEAT and ARM repeat proteins bind to fatty acyls conjugated to agarose beads. (**a**) Schematic diagram showing preparation of fatty acyl-conjugated ω-Aminohexyl-Agarose beads, adapted from E. Beck-García et. al., Anal Biochem, 2013. (**b**) Representative silver staining results of pull-down with acyl conjugated beads and recombinant HEAT or ARM repeats. BSA included as positive control for long-chain fatty acid binding, and AF10 PZP domain as negative control. Full-length gels are presented in Supplementary Fig. S2. (**c**) Normalized quantification of silver staining intensity of gels presented in 3b. (**d**) Percentage of enriched proteins containing HEAT or ARM repeats from C10:0 vs C2:0 and/or C14:0 vs C2:0 SILAC pull-down with HeLa whole cell lysate. (**e**) PANTHER GO-Molecular function annotation of the 79 HEAT and ARM proteins enriched in both C10:0 and C14:0 binding from SILAC pull-down experiments.

Next, we asked if the peptide backbone was dispensable for the interaction between HEAT and ARM repeat proteins and acyl moieties and if the interaction extended to fatty acyl chain lengths besides 14 carbons. We generated a panel of fatty acyl conjugated beads with chain lengths spanning from two to 22 carbon atoms (Fig. 3a, method adapted from^21^). We used the HEAT repeat protein 2AAB, and the ARM repeats of β-catenin and APC (Adenomatous polyposis coli) as models for this experiment. Given that GST has been previously shown to bind long-chain free fatty acids^22,23^, we cleaved the GST tags from the recombinant proteins with PreScission proteinase in these experiments. Interaction between recombinant proteins and acyl-beads was detected by silver staining (Fig. 3b, Supplementary Fig. S2)). A fatty acid carrier protein bovine serum albumin (BSA) was used as a positive control, and the PZP domain of AF10, a reader for unmodified lysine 27 of histone H3^24^, as a negative control. As expected, BSA binds to long-chain fatty acyl beads, which matches its known activity^25^ and the AF10 PZP domain does not bind to any fatty acyls. The ARM repeats of β-catenin bound a range of acyl groups from hexanoic acyl (C6:0) to myristic acyl (C14:0) with strongest binding to decanoic acyl (C10:0) and gradually weaker interaction as chain length increased or decreased from ten carbons. The ARM domain of APC also exhibited peak binding at C10:0 but with a sharp drop-off of interaction at C8:0 and a much more gradual decrease up through C22:0. 2AAB bound to C8:0 through C22:0 with a slight peak at C12:0. Quantification of normalized fold change in silver staining intensity is shown in Fig. 3c. These results demonstrate that HEAT and ARM repeat proteins bind to medium- and long-chain free fatty acyl moieties.

To ask whether fatty acyls interact with HEAT and ARM repeat proteins expressed in cells in an unbiased manner, we used decanoic (C10:0) and myristic (C14:0) beads as baits for pull-down experiments with acetic (C2:0) beads as the control pull-down and using stable isotope labeled HeLa whole cell extracts as described above for peptides (see Fig. 1a). In the C10:0 experiment, 51 of 532 enriched proteins were annotated HEAT and ARM repeat proteins (Supplementary Table S3). This is an approximate 1.7-fold increase in the proportion of HEAT and ARM proteins compared to the human proteome with an associated *p*-value of 6.75E-5 by Chi-Square test (Fig. 3d, Supplementary Table S4). In the C14:0 experiment, 55 of 491 enriched proteins were HEAT and ARM repeat proteins (Supplementary Table S5), representing a ~2-fold enrichment as compared to the proteome with a *p*-value of 6.92E-8 (Fig. 3d, Supplementary Table S4). Notably, β-catenin was enriched in the C10:0 pull-down (Supplementary Table S3). We used the PANTHER classification system to assess annotated molecular function GO-terms of the 79 unique HEAT and ARM proteins enriched in the C10:0 and C14:0 experiments and found a relatively widespread pattern (Fig. 3e). This finding suggests that the HEAT and ARM proteins that can bind to medium- and long-chain acyl molecules are involved in diverse cellular processes.

### HEAT and ARM repeat proteins bind fatty acyl molecules in cells

**Figure 4:**
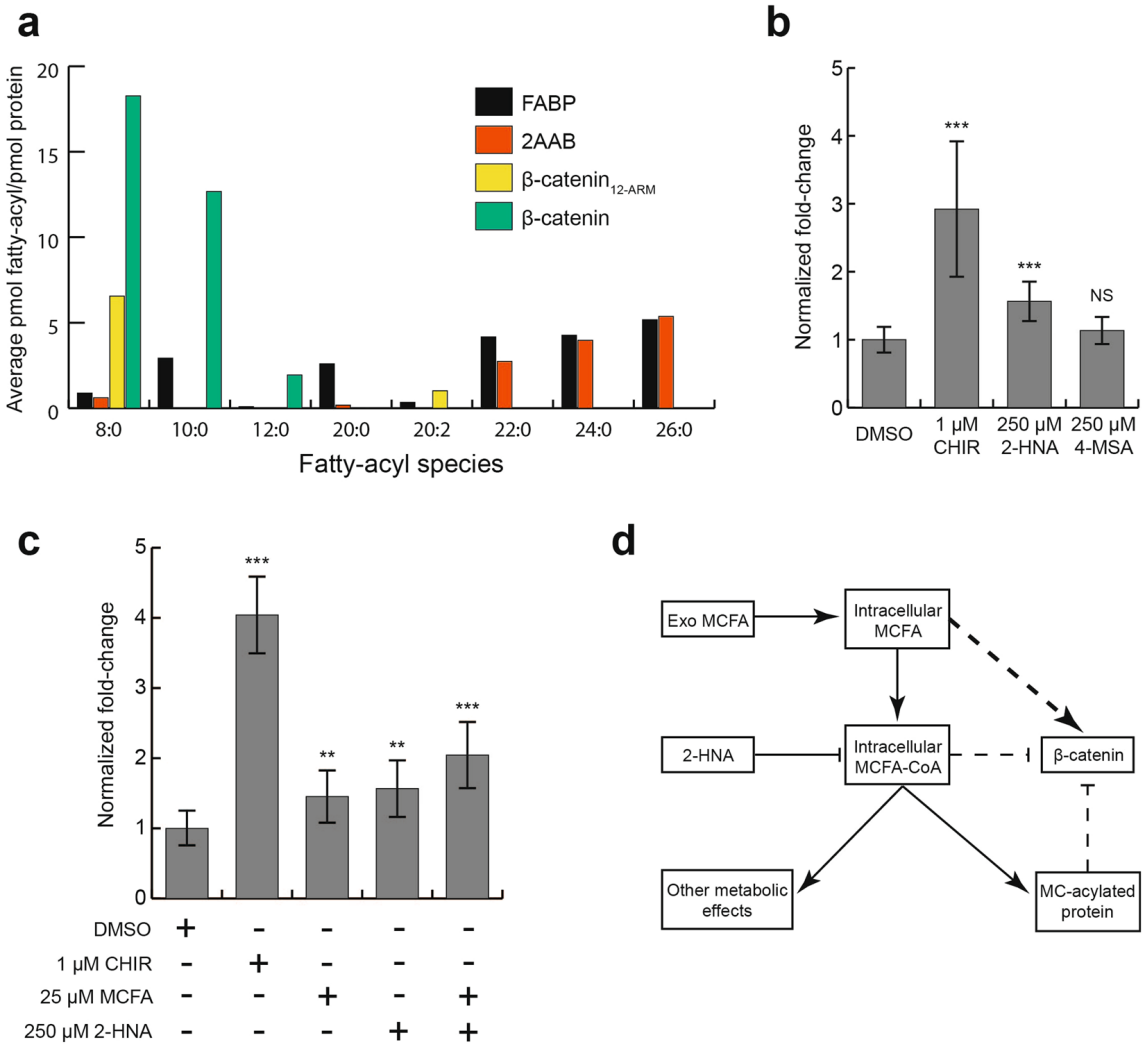
Fatty acids co-purify with HEAT and ARM repeat proteins in mammalian cells and exogenous medium-chain fatty acids alter β-catenin transactivation activity. (**a**) GC-MS identified and quantified fatty acids co-purified with HEAT and ARM repeat proteins expressed in 293T cells (β-catenin, 2AAB, and FABP are full-length). Fatty acids are normalized to protein molarity. (**b**) TOPflash luciferase reporter assay shows increase of β-catenin transactivation activity in presence of medium-chain fatty-acyl CoA synthase inhibitors 2-HNA (*p* = 2.49E-04) and 4-MSA (*p* = 6.70E-01) compared to DMSO control. CHIR99021 is a GSK3β inhibitor, included as a positive control (*p* = 3.57E-04). (**c**) TOPflash luciferase reporter assay shows increase in β-catenin transactivation activity in presence of 25 μM MCFA (~8.3 μM each of C8:0, C10:0, and C12:0, *p* = 9.17E-03), 250 μM 2-HNA (*p* = 3.21E-03), and the combination (*p* = 7.10E-05) compared to DMSO control, with CHIR99021 as positive control (*p* = 7.69E-09). Error bars in (**c**,**d**) are standard deviations of 3 independent biological replicates, with 3 technical replicates each. **p < 0.01, ***p < 0.001; un*p*aired two-tailed Student’s t-test. (**d**) Proposed model for activation of β-catenin transcriptional activity through exogenous MCFA and 2-HNA treatments shown in (**c**,**d**). β-catenin activity is promoted by free MCFA and/or inhibited by MCFA-CoA or MCFA-modified proteins.

We next asked whether fatty acyl molecules co-purify with HEAT and ARM repeat proteins in mammalian cells. 2AAB, the 12-ARM repeats of β-catenin, full-length β-catenin, fatty acid binding protein (FABP) as positive control, and the empty overexpression vector as negative control were transiently transfected in 293T cells and associated total fatty acids were determined by gas chromatography and mass spectrometry (GC-MS)^26^. Samples were subjected to alkaline-based hydrolysis, which cleaves off fatty acids attached via an ester bond. Thus, a given fatty acid signal might come from either the free fatty acid, the fatty acid ester, or both that co-purified with the bait proteins. The fatty acid signals in the empty vector sample were treated as background and subtracted from the signals in the other conditions. C16:0 and C18:0 are common contaminants in buffers and plasticware, and their signals were high in the background samples, so they were excluded from further analysis. Each fatty acyl signal was then normalized to molar quantities of the purified proteins. β-catenin co-purified with a high molar ratio of C8:0, C10:0 and C12:0 relative to the controls (Fig. 4a), closely matching the binding selectivity seen in the *in vitro* acyl-bead pull-down experiment in Fig. 3b. However, the 12-ARM repeats of β-catenin only co-purified with C8:0 and unexpectedly C20:2 (eicosadienoic acid) (Fig. 4a), suggesting that 12 ARM repeats alone may not be sufficient to recapitulate the acyl binding properties of the full-length protein under more physiological conditions. Long-chain fatty acids co-purified with 2AAB (Fig. 4a), which also matches its binding profile in the *in vitro* acyl-bead pull-down experiment in Fig. 3b. There was also a low C8:0 signal detected in the 2AAB samples (Fig. 4a), which was not detected in acyl-bead pull-down with recombinant 2AAB protein possibly because of the difference in sensitivity of the two assays. C20:0 through C26:0 co-purified with FABP (Fig. 4a), which matched previously reported binding activity of the protein^27^. The identification of endogenous fatty acids and/or fatty acid esters that interact with HEAT and ARM repeat proteins in cells suggests the intriguing possibility that HEAT and ARM repeat proteins might act as sensors of intracellular fatty acyls to fine tune cognate protein function.

### Targeting MCFA metabolism alters transactivation activity of the ARM repeat protein β-catenin

Intracellular concentrations of short- and medium-chain fatty acids are thought to closely match the composition of the media in which they are grown because of free diffusion of the molecules through the cell membrane^28^. Before cells can make use of fatty acids in metabolism or protein acylation pathways, these molecules must be activated through esterification to Coenzyme-A. We reasoned that manipulating medium-chain fatty acid (MCFA) metabolism might modulate β-catenin transactivation activity, giving insight into the functional consequence of interactions between medium-chain fatty acyls and the ARM repeats of β-catenin. To this end, we used a luciferase-based β-catenin reporter assay^29,30^. Two structurally distinct small molecules, 2-Hydroxy naphthoic acid (2-HNA) and 4-Methylsalicylic acid (4-MSA)^31^, were used to disrupt MCFA-CoA synthesis, which is predicted to reduce intracellular MCFA-CoA levels. Experiments were conducted in HT1080 cells, which express wild-type β-catenin and have low basal β-catenin transactivation activity. As expected, the positive control compound that activates β-catenin, the GSK-3β inhibitor CHIR99021, significantly increased β-catenin transactivation activity (Fig. 4b,c)^32^. 2-HNA treatment moderately, though significantly, increased β-catenin transcriptional activity, while 4-MSA showed a modest, but not statistically significant trend towards increased activity (Fig. 4b). This may be related to the relatively lower inhibitory activity of 4-MSA as compared to 2-HNA seen in *in vitro* assays^31^. We note that poor solubility of the compounds in cell culture media prevented us from testing higher concentrations.

Inhibition of MCFA-CoA synthesis could potentially cause accumulation of free MCFAs and/or depletion of MCFA-CoA for downstream metabolism. To distinguish between these possibilities, we supplemented cell media with exogenous MCFAs. Treatment with 25 μM of MCFAs containing equal molar amounts of C8:0, C10:0 and C12:0 caused a modest increase in β-catenin transcriptional activity (Fig. 4c). Additionally, MCFAs combined with 2-HNA further increased β-catenin transcriptional activity (Fig. 4c). These results suggest that the increased β-catenin transcriptional activity may be caused by excess free MCFAs in cells, and/or reduced inhibition from MCFA-CoAs or MCFA-modified proteins (Fig. 4d). Together with our previous data showing direct interaction between the ARM repeats of β-catenin and MCFA moieties, these results support the hypothesis for regulation of β-catenin via direct interactions with intracellular medium-chain fatty acyls, revealing a potential novel link between lipid metabolism and the β-catenin pathway.

## Discussion

We have identified HEAT and ARM repeats as general *in vitro* reader domains of medium- and long-chain fatty acyl groups, with the potential that different HEAT and ARM proteins may have distinctive binding profiles. For example, the β-catenin ARM repeats appear to bind better to K-modified versus N-terminal modified myristoylated proteins (see Fig. 2b). We have also provided evidence that alteration of intracellular MCFA and MCFA-CoA levels impacts, albeit modestly, the transactivation activity of the important ARM repeat protein β-catenin. β-catenin is the major downstream effector of Wnt signaling and plays important roles in the development and progression of various cancers^33^. β-catenin is actively generated and constantly degraded in the absence of Wnt signaling. Upon Wnt activation, the degradation of β-catenin is disrupted, which causes the accumulation of β-catenin and ultimately its nuclear translocation. This in turn leads to nuclear β-catenin binding to TCF/LEF transcription factors and its transactivation of target genes. We found that both the supplementation of MCFAs and the inhibition of MCFA-CoA synthesis increase β-catenin transactivation activity, suggesting a potential, more general hypothesis that is not proven here and only raises the idea that HEAT and ARM repeat proteins, like β-catenin, may under certain circumstances act as sensors of cellular fatty acyl metabolism. We at present do not know whether such a theoretical function is mediated directly by MCFAs acting as signaling molecules that modulate ARM/HEAT proteins or through ARM/HEAT repeats binding to acylated proteins.

The HEAT and ARM repeat proteins identified in our proteomic studies span a wide range of biological processes, including nuclear transport, DNA regulation, and cytoskeleton regulation, among others. Future work may be able to investigate how these segregated subcellular processes are affected by fatty acids and how cells respond to fatty acid metabolic changes. While the form of acyl molecules that bind to HEAT and ARM repeat proteins in cells (e.g. free fatty acids, acyl-CoA, or acyl-modified proteins) remains unclear, an exciting possibility exists where individual HEAT and ARM proteins show functionally relevant selectivity for (1) the type of acyl group in addition to chain-length, (2) the level of saturation, and (3) perhaps attachment to protein in order to exert unique biological effects. As novel techniques are developed for the study of medium- and long-chain fatty acyl molecules, we will be able to further interrogate how cellular fatty acid metabolism may impact diverse biological processes through the lens of HEAT and ARM repeat proteins.

## Methods

### Peptides, protein sequences and plasmids

All peptides were synthesized on PTI Symphony automated synthesizer. The peptides containing K(Biotin) were synthesized on PEG based resin (ChemMatrix Rink amide resin), K(Biotin) residue was introduced using Fmoc-Lys(Biotin)-OH. The peptides with Peg-Biotin at C-terminus were synthesized using NovaTag Fmoc-Peg-Biotin resin. The N-terminal alanine was introduced as Boc-Ala-OH. K(acyl) residues were introduced using orthogonaly protected lysine - Fmoc-Lys(Mtt)-OH, After peptide synthesis Mtt group was removed by treatment with 2% trifluoroacetic acid, 4% triisopropylsilane in dichloromethane (3 × 20 min) and deprotected lysine residue was the acylated by treatment with acyl anhydrides and diisopropylethylamine (1:2 molar ratio) in DMF. The only exception was 3-hydroxybutyryl group, it was introduced using 3-hydroxybutiric acid, HATU, and diisopropylethylamine (1:1:3 molar ratio) in NMP. The lysine acylation was confirmed with ninhydrin test. Peptides were cleaved from the resin by 2 h treatment with 2.5% triisopropylsilane, 2.5% water in trifluoroacetic acid and precipitated with a cold diethyl ether. After precipitation peptides were separated by centrifugation washed 3 x with diethyl ether, air dried, dissolved in 50% acetonitrile and lyophilized. Crude peptides were purified by preparative HPLC and peptide composition was confirmed by MALDI-TOF mass spectrometry. Peptide sequences are: H3 1–20 series (acyl modifications on lysine 9), ARTKQTARK(acyl)STGGKAPRKQL-K(Biotin); H3 15–34 series (acyl modifications on lysine 27), APRKQLATKAARK(acyl)SAPSTGG-Peg-Biotin; BID 61–70 unmodified, GNRSSHSRLG-Peg-Biot; BID 61–70 N-myristoylated, Myr-GNRSSHSRLG-Peg-Biot. Recombinant HEAT or ARM repeat protein sequences: GCN1 7-HEAT repeats, 1402–1715 aa; DNAPK single HEAT repeat, 276–331 aa; IMB1 single HEAT repeat, 396–459 aa; 2AAB full length, 1–601 aa; β-catenin 12-ARM repeats, 135–664 aa; APC 7-ARM repeats, 440–783 aa. pGEX-6p-1 (GE Healthcare) was used to express the proteins as N-terminal fusions with glutathione-S-transferase (GST). For mammalian expression, pcDNA3.1 (Invitrogen) were used and an N-terminal FLAG tag was added to the proteins to enable affinity purification.

### Cell culture

Both HEK293T (ATCC), and HT1080 (ATCC) were cultured in Dulbecco’s modified Eagle’s Medium (DMEM, Life Technologies) supplemented with 10% fetal bovine serum, 2 mM L-glutamine and penicillin–streptomycin (Life Technologies).

For stable isotope labeling, Hela cells were cultured in DMEM medium for SILAC (Thermo fisher, cat # 88364) supplemented with 10% dialyzed FBS (Sigma, F0392) and stable isotope labeled lysine and arginine (light: 12 C and 14 N; heavy: 13 C and 15 N; 50 mg each for 500 mL medium). 13 C and 15 N labeled lysine and arginine are from Silantes (# 211604102 and # 201604102). In addition, 115 mg of proline (AppliChem) was supplemented to 500 mL medium to increase the incorporation of exogenous lysine and arginine. Cells were cultured for at least five cell divisions to achieve a near 100% labeling of the proteins.

### Peptide pull-down with stable isotope labeled Hela nuclear extracts

Nuclear extract was prepared by lysing cells in hypotonic buffer and salt extraction of nuclei. Briefly, cells were lysed in hypotonic lysis buffer containing 10 mM KCl, and intact nuclei were separated from cytoplasmic proteins by centrifugation. The nuclear proteins were extracted with high salt (420 mM KCl) buffer. The protein concentration was determined with Bradford assay.

All peptides were C-terminal biotinylated so that peptide pull-down experiments could be carried out with streptavidin beads (Dynabeads streptavidin T1, Thermo fisher, 65602). 10x excess of peptides were used to bind and saturate the streptavidin beads. The peptide-bound beads (40 uL slurry) were incubated with 1 mg Hela nuclear extracts. Two sets were done for each experiment, Forward (unmodified-light, acylated-heavy) and Reverse (unmodified-heavy, acylated-light). After incubation, unmodified and acylated beads in the same set of experiments were washed and combined. The bound proteins were eluted with 2x SDS sample buffer (100 mM Tris-HCl pH6.8, 4% SDS, 12% glycerol, 2% β-mercaptoethanol, 0.008% bromophenol), separated with the bait peptides on SDS-PAGE, and subjected to trypsin digestion and LC-MS/MS analysis.

### Liquid chromatography and tandem mass spectrometry (LC-MS/MS)

Proteins from SILAC pull-down experiments were separated by SDS-PAGE and subjected to in-gel tryptic digest. Gel bands containing proteins were cut, treated with 10 mM DTT and alkylated with 50 mM iodoacetamide before trypsin (Promega) digestion. Digested peptides were extracted from the gel with 50% acetonitrile and 75% acetonitrile in 0.1% formic acid successively and dried in a SpeedVac. Peptides were desalted with a C18 stagetip (Thermo Scientific). Peptides were separated by high pressure liquid chromatography (HPLC) using an Dionex Ultimate 3000 LC-system (Thermo Scientific) and analyzed with an Orbitrap Elite mass spectrometer (Thermo Scientific). Data acquisition was executed in data dependent mode with full MS scans acquired in the Orbitrap mass analyzer and selection of top 20 ions with dynamic exclusion followed by collision induced dissociation (CID) analysis of fragment ions in the ion trap with rapid scan rate.

Protein identification and quantification were done with MaxQuant version 1.5.2.8. For proteins with no heavy/light ratio reported, an in-house program was used to check all peptide signals. Peptides with a MaxQuant peptide ratio of acyl/unmodified >2 or an infinitely large peptide intensity ratio (acyl/unmodified, when unmodified intensity was zero) were considered enriched. Proteins with at least three enriched peptides and no peptide change in the opposite direction were considered enriched and added to the final enrichment list.

### Peptide pull-down with recombinant proteins

GST-tagged recombinant proteins are expressed in *E. coli* BL21 strain at 16 °C for 20 hours in the presence of 0.1 mM IPTG (Sigma), purified with glutathione sepharose (GE Healthcare) in protein lysis buffer (50 mM Tris-HCl pH8.0, 150 mM NaCl, 0.1% Nonidet P-40, 0.5 mM PMSF) and eluted with 10 mg/mL reduced glutathione (Sigma) in 100 mM Tris-HCl (pH 8.0). Peptide pull-downs with recombinant proteins were done in the same manner as with the Hela cell lysate. 1 ug recombinant proteins were used for binding with 10 uL peptide-saturated streptavidin beads. After pull down, proteins were detected by western blotting with an in-house antibody against the GST tag.

### Fatty acyl-conjugated agarose and pull-down experiment

Fatty acids with chain length from two carbons to 20 were conjugated to ω-Aminohexyl–Agarose (Sigma/Millipore, cat# A6017) following a method developed in^21^ with modifications. Briefly, 46 μmol fatty acid anhydride (Sigma) and 92 μmol DIEA (N,N-Diisopropylethylamine, Sigma cat# 387649) were added to 0.5 mL ω-Aminohexyl–Agarose bead volume in 1 mL dimethylformamide (Sigma). Reactions were carried out at 65 °C with stirring for 1 hour. The beads were washed five times with dimethylformamide. To test the completeness of the blocking of the amine group on the agarose beads by fatty acid, a ninhydrin test (AnaSpec) was carried out on aliquots of conjugated beads. Reactions were repeated until the beads showed no blue color in the ninhydrin test.

Fatty acid-conjugated agarose beads were then washed twice in 1X DPBS (Dulbecco’s phosphate-buffered saline, Gibco), and stored in 1X DPBS and kept at 4 °C for no more than two weeks. Pull-down experiments with recombinant proteins were carried out in buffer (20 mM Tris-HCl pH 8.0, 137 mM NaCl, 2 mM EDTA, 10% glycerol, 0.5% Brij O10). GST tags were cleaved from recombinant proteins with in-house made PreScission proteinase during purification. 3 ug of recombinant proteins and 20 uL of fatty acid-conjugated agarose beads (50% slurry) were incubated for 1 hr at room temperature. After pull-down, beads were washed three times in the same buffer and 50 uL of 5X SDS loading buffer was added, and beads were then boiled at 100 °C for 5 minutes before resolution through SDS-PAGE and detection by silver staining (SilverQuest, Invitrogen). Silver staining intensities of the protein bands were quantified using ImageJ, and values from empty beads were subtracted from all other lanes, followed by normalization to the inputs. Any negative values were set to zero.

For fatty acyl beads pull-down experiments with stable isotope labeled Hela cell lysate, the same procedure was used as with the recombinant proteins. The binding proteins were subjected to LC-MS/MS analysis following the same procedure as described for the peptide pull-downs.

### Gas chromatography and mass spectrometry (GC-MS) analysis of fatty acids co-purified with HEAT and ARM repeat proteins

Proteins with an N-terminal 1xFLAG-tag were overexpressed in 293T cells. 10 ug of pcDNA3.1-FLAG empty vector or the protein in the same vector (2AAB, β-catenin full length, β-catenin 12-ARM repeats (135–664 aa), and FABP) were transfected with TransIT-293 transfection reagent (Mirus) according to manufacturer instructions. 48 hr after transfection (two 15-cm plates for each protein), cells were lysed in IP buffer (50 mM Tris-HCl pH 8.0, 200 mM NaCl, 1 mM EDTA, 10% glycerol, 0.1% Nonidet P-40, Roche proteinase inhibitor cocktail) and FLAG-tagged proteins were purified with 10 uL anti-FLAG M2 magnetic beads (Sigma). Proteins were eluted with 0.5 mg/mL 3xFLAG peptides in TBS buffer (50 mM Tris-HCl pH 8.0, 150 mM NaCl). A proportion of the proteins along with known amounts of BSA were detected on an SDS-PAGE gel by Coomassie staining, and ImageJ was used to measure the staining intensity of protein bands. A standard curve was established based on BSA protein amounts and Coomassie staining intensities, and the concentrations of eluted proteins were calculated based on the standard curve. At least 3 pmol of each protein were subjected to total fatty acid analysis at the UCSD Lipidomics Core. Alkaline based hydrolysis was performed prior to extraction of fatty acids. Fatty acids were prepared and analyzed as described in^26^.

To remove background signal, each fatty acid (FA) signal from the empty vector sample was subtracted from other samples and resulting negative values were forced to zero. Sample values (pmol FA/mL) were then normalized to the protein concentrations of each purified protein (pmol protein/mL), yielding the proportion of pmol FA:pmol protein. Results from two biological replicates were then averaged.

### TOP-flash luciferase assay

The lentiviral luciferase-based vector contains 7-TCF binding sites upstream of firefly luciferase and puromycin N-acetyltransferase (hence 7TFP, AddGene #24308). Virus particles were produced by co-transfection of HEK 293T cells with pCMV-VSVG (AddGene #8454) and pCMV-dR8.2 (AddGene #8455). HT1080 cells were transduced with virus and 4 µg/mL polybrene (Millipore) followed by selection starting at 48 hours with 2 µg/mL puromycin (Sigma). Cells were grown in selection media for at least a week before plating for experiments. Generally, 50 thousand cells in 100 uL were seeded in triplicate in a black, clear bottom 96-well plate for each condition tested. 3 hours post-seeding, media was replaced with fresh media and small molecules or exogenous medium-chain fatty acids were added. Cells were treated for 24 hours, except for CHIR99021, which was added 6 hours prior to luciferase detection. Steady-Glo Luciferase Assay Substrate (Promega) was added to each well and luminescence was then measured in a Synergy H1 microplate reader (BioTek). Signals from the three technical replicate wells were averaged for further analysis. At least three biological replicates were performed for each condition.

### Panther GO-term analysis

Briefly, the annotated HEAT and ARM proteins that were enriched in the C10:0 and C14:0 lipid bead SILAC pull down experiments were combined and submitted to the PANTHER classification system for functional classification enrichment analysis using Bonferroni correction for multiple testing^34–36^.

## Supplementary information

Supplementary Figures 1 and 2

Supplementary Table S1

Supplementary Table S2

Supplementary Table S3

Supplementary Table S4

Supplementary Table S5

## Data Availability

The mass spectrometry datasets generated and/or analysed during the current study are available in the ProteomeXchange Consortium via the PRIDE^37^ partner repository with dataset identifiers PXD015233 and PXD015272. All other data generated or analysed during this study are included in this published article (and its Supplementary Information files), or are available from the corresponding author upon reasonable request.
