# Supplementary material for: Binding to medium and long chain fatty acyls is a common property of HEAT and ARM repeat modules
Source: Sci Rep. 2019 Oct 2;9:14226. doi: 10.1038/s41598-019-50817-6 (PMC6775327; doi:10.1038/s41598-019-50817-6)
Supplement: Supplementary file 1 — Supplementary Figures 1 and 2 [file 41598_2019_50817_MOESM1_ESM.pdf]

Supplementary information

Binding to medium and long chain fatty acyls is a common property of HEAT and ARM repeat modules

Tie-Mei Li, John P. Coan, Krzysztof Krajewski, Lichao Zhang, Joshua E. Elias, Brian D. Strahl, Or Gozani and Katrin F. Chua

Supplementary Fig. S1. Scatter plots showing the protein quantification results of all SILAC peptide pull-downs.

Supplementary Fig. S2: Full-length blots and gels for Figure 2a, 2b and 3b.

Separate Excel documents:

Supplementary table S1: Protein identification and quantification results of H3K9 acyl-vs-unmodified peptide pull-down with stable isotope labeled Hela nuclear extracts.

Supplementary table S2: Protein identification and quantification results of H3K27 acyl-vs-unmodified peptide pull-down with stable isotope labeled Hela nuclear extracts.

Supplementary table S3: Proteins enriched with H3K9myr peptide, K3K27myr peptide, C10:0, and C14:0 agarose beads.

Supplementary table S4: Chi-square tests for enrichment of HEAT and ARM repeat proteins in H3K9myr peptide, K3K27myr peptide, C10:0, and C14:0 agarose bead pull-downs.

Supplementary table S5: Protein identification and quantification results of C10:0-vs-C2:0 and C14:0-vs-C2:0 agarose bead pull-down with stable isotope labeled Hela cell extracts.

Figure S1

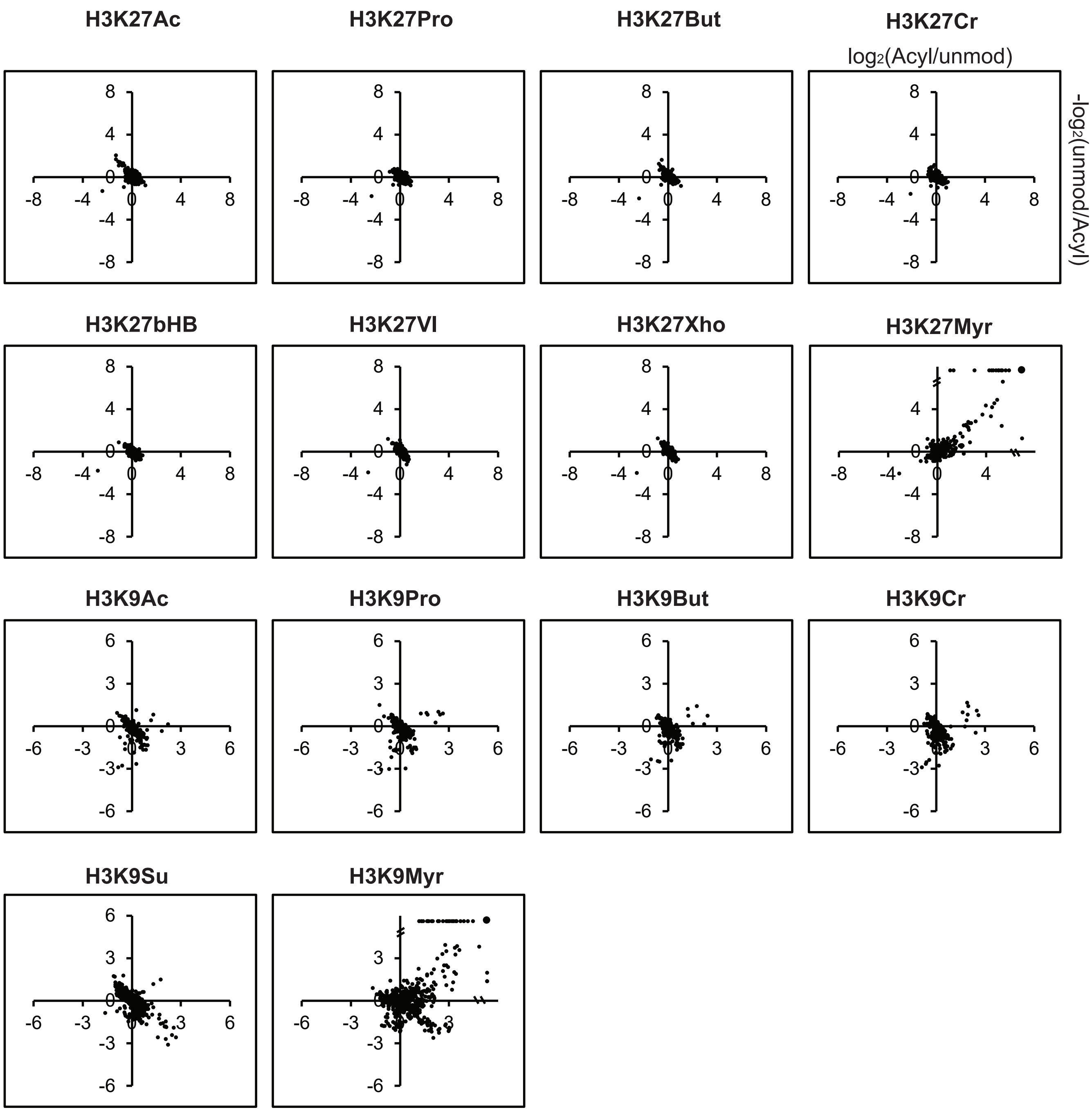
H3K27Ac
H3K27Pro
H3K27But
H3K27Cr
log2(Acyl/unmod)
-log2(unmod/Acyl)
H3K27bHB
H3K27Vl
H3K27Xho
H3K27Myr
H3K9Ac
H3K9Pro
H3K9But
H3K9Cr
H3K9Su
H3K9Myr

Figure S2

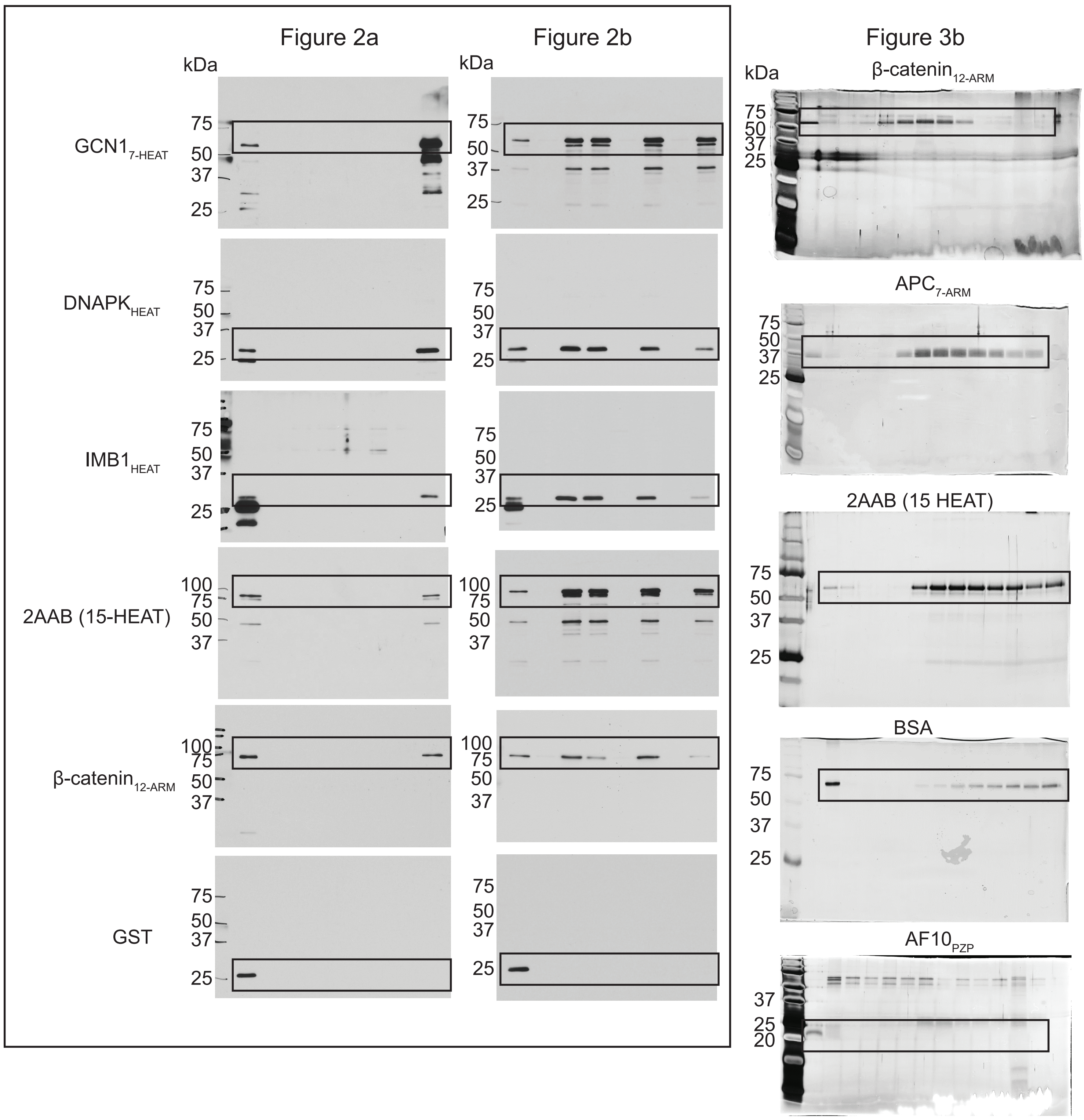

Figure 2a
Figure 2b
Figure 3b
kDa
GCN1$_{7\text{-HEAT}}$
DNAPK$_{\text{HEAT}}$
IMB1$_{\text{HEAT}}$
2AAB (15-HEAT)
β-catenin$_{12\text{-ARM}}$
GST
APC$_{7\text{-ARM}}$
2AAB (15 HEAT)
BSA
AF10$_{\text{PZP}}$
100
75
50
37
25
20
